# Host Range Expansion of *Shigella* Phage Sf6 Evolves through Point Mutations in the Tailspike

**DOI:** 10.1128/jvi.00929-22

**Published:** 2022-07-27

**Authors:** Sundharraman Subramanian, John A. Dover, Kristin N. Parent, Sarah M. Doore

**Affiliations:** a Department of Biochemistry and Molecular Biology, Michigan State University, East Lansing, Michigan, USA; b Department of Microbiology and Cell Science, University of Florida, Gainesville, Florida, USA; University of Kentucky College of Medicine

**Keywords:** bacteriophage evolution, bacteriophages, experimental evolution, protein structure-function, structural biology, virus-host interactions

## Abstract

The first critical step in a virus’s infection cycle is attachment to its host. This interaction is precise enough to ensure the virus will be able to productively infect the cell, but some flexibility can be beneficial to enable coevolution and host range switching or expansion. Bacteriophage Sf6 utilizes a two-step process to recognize and attach to its host Shigella flexneri. Sf6 first recognizes the lipopolysaccharide (LPS) of S. flexneri and then binds outer membrane protein (Omp) A or OmpC. This phage infects serotype Y strains but can also form small, turbid plaques on serotype 2a_2_; turbid plaques appear translucent rather than transparent, indicating greater survival of bacteria. Reduced plating efficiency further suggested inefficient infection. To examine the interactions between Sf6 and this alternate host, phages were experimentally evolved using mixed populations of S. flexneri serotypes Y and 2a_2_. The recovered mutants could infect serotype 2a_2_ with greater efficiency than the ancestral Sf6, forming clear plaques on both serotypes. All mutations mapped to two distinct regions of the receptor-binding tailspike protein: (i) adjacent to the LPS binding site near the N terminus; and (ii) at the distal, C-terminal tip of the protein. Although we anticipated interactions between the Sf6 tailspike and 2a_2_ O-antigen to be weak, LPS of this serotype appears to inhibit infection through strong binding of particles, effectively removing them from the environment. The mutations of the evolved strains reduce the inhibitory effect by either reducing electrostatic interactions with the O-antigen or increasing reliance on the Omp secondary receptors.

**IMPORTANCE** Viruses depend on host cells to propagate themselves. In mixed populations and communities of host cells, finding these susceptible host cells may have to be balanced with avoiding nonhost cells. Alternatively, being able to infect new cell types can increase the fitness of the virus. Many bacterial viruses use a two-step process to identify their hosts, binding first to an LPS receptor and then to a host protein. For *Shigella* virus Sf6, the tailspike protein was previously known to bind the LPS receptor. Genetic data from this work imply the tailspike also binds to the protein receptor. By experimentally evolving Sf6, we also show that point mutations in this protein can dramatically affect the binding of one or both receptors. This may provide Sf6 flexibility in identifying host cells and the ability to rapidly alter its host range under selective pressure.

## INTRODUCTION

Bacteriophages, also known as phages, are viruses that infect bacteria. These viruses are diverse, ancient, and ubiquitous (1). Phages persist in a variety of conditions, within fluctuating or stable environments, and likely encounter numerous different types of cells. Like all viruses, a fundamental step in the phage infection cycle is recognition of a host, including the ability to discriminate between susceptible and nonsusceptible hosts. For many tailed phages infecting Gram-negative bacteria, this often involves a two-step process: first, reversible binding to a primary receptor; second, irreversible binding to a secondary receptor (2, 3). The primary receptor is often the lipopolysaccharide (LPS) that extends from the cell surface and coats the membrane. The secondary receptor is more limited, such as one or two types of proteins embedded in the membrane. Using these mechanisms, bacteriophage host range can be broad, with a phage able to infect multiple species or genera; or narrow, with a phage being restricted to hosts with specific LPS chemistry.

Members of the genus *Shigella* are Gram-negative bacterial human pathogens causing bacillary dysentery, with infection usually linked to contaminated food or water sources (4, 5). *Shigella* persists in environmental surfacewater, where it routinely emerges and infects over 250 million people annually and results in approximately 212 thousand deaths (6). Once ingested, the bacteria proliferate intracellularly in epithelial cells of the large intestine, causing severe diarrhea and dehydration (4, 7, 8). Of all *Shigella* species, S. flexneri is most frequently found in developing countries but has been responsible for outbreaks across the world, with different serotypes predominating different regions (5, 7). The serotype is determined by the repeating sugar units of the O-antigen and their subsequent glucosylation or acetylation, with Y being the simplest and least decorated type (9, 10). To date, over 50 O-antigen variants have been described for S. flexneri, with new serotypes and subtypes regularly emerging, often due to a combination of serotype-converting prophages and selection for virulence or immune evasion (9, 11, 12). This property has made vaccine development particularly challenging (13).

**FIG 1:**
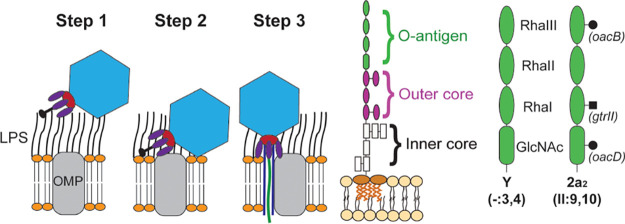
Overview of Sf6 virus attachment and infection (left), with the general structure of lipopolysaccharide (LPS) indicated in the center. Differences between the serotypes of host strains PE577 and CFS100, Y and 2a_2_, respectively, are shown (right). Modified from references 22, 39, 57.

Since attachment and genome ejection are the first stages of interaction between virus and host, determining how phages recognize a given serotype is critical to our understanding of phage biology and ecology, as is how they may switch between serotypes or expand into new serotypes. Shigella flexneri phage Sf6 is a member of the *Podoviridae* family and is closely related to phage P22. Using cryo-electron tomography, the steps involved in the initial contact between P22 and its host Salmonella enterica sv. Typhimurium have been visualized (14) and the structures of both P22 and Sf6 tail proteins have been resolved by various methods (3, 14–20). As part of the tail machinery, the tailspikes of P22 and Sf6 recognize and bind the O-antigen of LPS and then cleave the sugars using endorhamnosidase activity, bringing the phage closer to the host membrane (Fig. 1) (20, 21) In the case of Sf6, this lets an undetermined protein of the particle’s tail subsequently interact with outer membrane proteins (Omps) A or C as their secondary receptors (22, 23). In general, phage-mediated cleavage of LPS has been extensively studied and the structures and catalytic mechanisms are known for a variety of tailspike homologs (20, 24–29). It has also been determined that phage P22 interacts weakly with O-antigen derived from nonhost Salmonella enterica sv. Paratyphi, with the oligosaccharide occupying an energetically unfavorable conformation (30). For P22, LPS alone is sufficient to induce genome ejection *in vitro* (31). In phages where LPS is necessary and sufficient for genome ejection, a change from weak interactions between the phage tail and nonhost LPS to strong ones may be sufficient to confer an altered or broader host range. However, Sf6 requires both LPS and a secondary receptor to eject its genome *in vitro* (22, 23). This phage has been shown to use outer membrane protein A (OmpA), OmpC, and likely a third alternate protein receptor *in vivo* (22, 23). How the latter type of phage adapts to infect new hosts through interactions with both LPS and secondary receptors has not received much attention.

Phage Sf6 infects a narrow range of S. flexneri, being primarily limited to serotype Y hosts (32). Previous experimental evolution results also suggest that another podovirus, T7, reproducibly contracts its host range when passaged in the presence of multiple nonsusceptible hosts (33), suggesting specialization may be more favorable in multispecies environments. In this study, we experimentally evolved phage Sf6 in the presence of both susceptible serotype Y and nonsusceptible serotype 2a_2_ strains of S. flexneri and then isolated mutants with expanded host range. The O-antigen between serotypes Y and 2a_2_ differ by three modifications, as shown in Fig. 1. To capture as many potential routes of evolution as possible, we employed a high-throughput method to screen approximately 300 individually passaged populations. Our results show that the tailspike protein is the critical structure for mediating not only LPS interactions, but it also plays a role in interacting with the secondary receptor, particularly OmpC in this scenario. In addition, most phage-LPS interactions were previously thought of as reversible if the phage bound to a nonsusceptible host. Here we show that phage-LPS interactions can be detrimental to the phage. Rather than binding weakly or reversibly, bacterial LPS can bind in a way that inactivates the phage tail, rendering the particle no longer infectious. Similar to outer membrane vesicles, LPS may contribute to “herd immunity” in a mixed community of bacteria and phage, but with reduced cost. A more thorough understanding of these interactions between S. flexneri and its phages will therefore lead to understanding the ecology and evolution of S. flexneri persistence in the environment and facilitate the development of tools for treating infections or detecting its presence.

## RESULTS

### Mutations conferring broader host range map to the tailspike gene.

**FIG 2:**
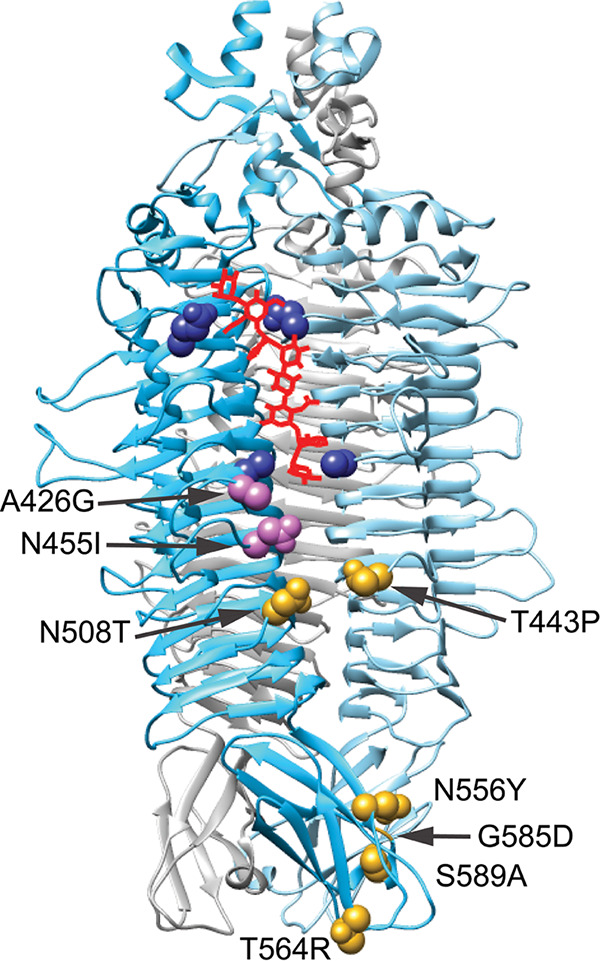
Crystal structure of the Sf6 tailspike trimer (PDB: 2VBM [20]), with the location of recovered mutations mapping to the indicated sites. A model of LPS is shown in red, with residues involved in LPS binding or catalysis highlighted in dark blue. Mutations recovered most frequently are in pink; all other mutations are in gold.

We initially attempted to experimentally evolve Sf6 using single-step selections on bacterial lawns or serial passages in liquid culture with only the new serotype 2a_2_ host CFS100. These attempts with nonsusceptible hosts alone were unsuccessful: no plaques were recovered, and all phage populations died out within the first few passages. To avoid this issue, our next trial in bulk liquid culture used a mixture of 90% nonsusceptible host (serotype 2a_2_ CFS100) and 10% susceptible host (serotype Y PE577). Using this method, we were able to isolate one mutant that could form plaques on CFS100. After whole genome sequencing, a single mutation was identified in gene 14 encoding the tailspike protein (gp14), which substituted asparagine 455 to isoleucine (N455I). Afterwards, to increase our ability to recover and detect mutants with expanded host range, we used deep-well 96-well plates and 0.5-mL volumes to propagate phage in parallel (see Fig. S1 for evolution scheme). At the end of each incubation period, the bacteria were lysed with chloroform so only the phages were passed to a fresh culture. Using this method, approximately 300 replicate populations were serially passaged, from which 34 isolates were found to infect the new host CFS100. The recovered mutants were plaque purified to ensure each phage was isogenic and then amplified to high titer for subsequent analysis. Initial characterization included whole genome sequencing of purified genomic DNA, which revealed that each mutant had only 1- or 2-bp changes in the entire ~40-kbp genome, which is consistent with previous Sf6 evolution schemes (34). As before, all mutations were in the Sf6 *gp14* gene, which encodes the tailspike protein. This suggested these point mutations were necessary and sufficient to confer the phenotype and that the tailspike is critical for determining host range. The tailspike protein is present as six trimers surrounding the portal and tail needle (represented by purple proteins in Fig. 1). Each trimer exhibits endorhamnosidase activity, responsible for cleaving the O-antigen repeating unit of LPS, with key catalytic residues found on different subunits that line the groove between their adjacent β-helices (Fig. 2, lower pair of blue amino acids) (20). Although 34 individual isolates were identified, these represent 8 nonsynonymous mutations and 9 genotypes. On the tailspike protein, these changes cluster around two distinct regions: one below the LPS binding site and the other at the distal tip of the protein (Fig. 2, pink and yellow). The changes highlighted in pink, A426G and N455I, arose the most frequently, representing 16 and 11 isolates, respectively (Table 1). These mutations were also found in tandem with other changes to produce four double mutants: A426G/N455I, A426G/N508T, A426G/T564R, and N455I/N556Y. The three remaining mutants were single nonsynonymous mutations: T443P, G585D, or S589A. The resulting amino acid substitutions do not appear to cause any significant protein folding defects, as both the titer of viral stocks and the ability to grow on the serotype Y host are indistinguishable from the ancestor phage.

**TABLE 1:** Nucleotide and amino acid changes of all serotype 2a_2_-infecting mutants, including the earliest passage in which they were identified and the total number of times they were isolated

Nucleotide change	Amino acid change	Earliest isolation	Total isolations
C14998G	A426G	6	16
C14998G/A15085T	A426G/N456I	4	1
C14998G/A15244C	A426G/N509T	17	1
C14998G/A15411G	A426G/T564R	17	1
A15048C	T443P	6	1
A15085T	N455I	4	11
A15085T/A15387T	N455I/N556Y	4	1
G15475A	G585D	7	1
T15486G	S589A	11	1

### Mutants are cold sensitive on the new host.

To determine how efficiently these mutants could infect the 2a_2_ serotype host, and under which parameters, a series of quantitative plaque assays were conducted. First, mutants were examined at various temperatures on both the serotype Y and serotype 2a_2_ hosts. All except two mutants were completely unable to form plaques at or below 25°C on CFS100 (Table 2). It was previously reported that S. flexneri produces greater levels of LPS and lower levels of OmpC at lower temperatures (35). Since OmpC was previously identified as a secondary receptor for ancestral Sf6 (22), we hypothesized that reduction of OmpC production at lower temperatures may have affected the plating efficiency for these tailspike mutants. The mechanism behind this cold sensitive (*cs*) phenotype was therefore investigated by making a series of genetic knockouts. These knockouts targeted either the bacterial LPS structure, outer membrane proteins A or C, or a combination of both. For LPS, knockouts of *waaL* and *gtrII* affect the O-antigen by either removing it entirely or by removing the glucosyl group of the 2a_2_ repeating unit, respectively.

**TABLE 2:** Efficiency of plating of ancestral Sf6 and mutants at different temperatures^*a*^

Phage genotype	Serotype Y		Serotype 2a		Serotype 2a/*ΔgtrII*	
	25°C	37°C	25°C	37°C	25°C	37°C
Ancestor	0.3 ± 0.2	1.0	<10^−6^	<10^−6^	0.3 ± 0.0	0.9 ± 0.1
A426G	0.4 ± 0.2	1.0	<10^−6^	0.6 ± 0.0	0.8 ± 0.2	0.7 ± 0.1
A426G/N455I	0.6 ± 0.3	1.0	0.1 ± 0.0	0.5 ± 0.1	0.5 ± 0.2	0.7 ± 0.2
A426G/N508T	0.5 ± 0.2	1.0	<10^−6^	0.5 ± 0.1	0.9 ± 0.6	0.8 ± 0.1
A426G/T564R	0.5 ± 0.4	1.0	<10^−6^	0.6 ± 0.1	0.9 ± 0.6	0.7 ± 0.1
T443P	0.6 ± 0.2	1.0	<10^−6^	0.7 ± 0.1	0.6 ± 0.0	0.9 ± 0.1
N455I	0.2 ± 0.1	1.0	<10^−6^	0.5 ± 0.1	0.8 ± 0.3	0.6 ± 0.1
N455I/N556Y	0.1 ± 0.0	1.0	0.1 ± 0.0	0.2 ± 0.1	1.0 ± 0.6	0.7 ± 0.2
G585D	0.1 ± 0.0	1.0	<10^−6^	10^−2^	1.1 ± 1.0	0.8 ± 0.2
S589A	0.5 ± 0.1	1.0	<10^−6^	10^−2^	0.9 ± 0.2	0.8 ± 0.2

a Gray shading indicates low efficiency of plating values (≤10^−2^), with darker gray corresponding to lower efficiency.

As shown in Table 3, *ΔwaaL* prevents plaque formation of any phage isolate in either background, consistent with previous results showing that Sf6 requires O-antigen to attach to its host (20). Removing *gtrII* in the CFS100 background restored the ability of all mutants to form plaques at all temperatures, including 25°C, suggesting this glucosyl group is necessary to confer resistance. Conversely, removing *ompC* reduced plaque formation on the new host for all mutants, although this phenotype was most severe for G585D and S589A. These two mutants showed reduced efficiency of plating in both CFS100/*ΔompA* and CFS100/*ΔompC* strains, with a 1- to 2-fold log further reduction than the other mutants on CFS100/*ΔompC*. Even in the original serotype Y host, plaque formation of S589A is reduced 10-fold in the *ΔompC* strain, again indicating that this mutant is more sensitive to a lack of OmpC. The more severe phenotype of these mutants suggests the region these mutations map to, the distal tip of the tailspike, mediates interactions with these proteins. The changes in electrostatics, especially of the single mutant G585D, are complementary and consistent with recent work identifying charged amino acid side chains in the surface loops of OmpC as being important for *Shigella* phage attachment (36). In addition, since the OmpC protein sequence is identical between these two strains, the difference in plating efficiency can be attributed to the differences in the LPS. Although T564R represents an alternative mutation at this distal tip of the tailspike, it was found only in tandem with A426G; thus, it is difficult to speculate on the phenotype of a T564R single mutant. We hypothesize this mutant is not as reliant on OmpC due to the additional altered LPS interactions conferred by A426G.

**TABLE 3:** Efficiency of plating of ancestral Sf6 and mutants on strains with or without the indicated receptors at 37°C^*a*^

	Serotype Y				Serotype 2a				Serotype 2a/*ΔgtrII*		
Phage	−	*ΔwaaL*	*ΔompA*	*ΔompC*	–	*ΔwaaL*	*ΔompA*	*ΔompC*	–	*ΔompA*	*ΔompC*
Ancestor	1.0	<10^−6^	1.3 ± 0.1	0.5 ± 0.2	<10^−6^	<10^−6^	<10^−6^	<10^−6^	0.9 ± 0.1	0.9 ± 0.2	0.3 ± 0.2
A426G	1.0	ND	1.2 ± 0.1	0.4 ± 0.2	0.6 ± 0.0	ND	0.6 ± 0.1	10^−2^	0.7 ± 0.1	1.3 ± 0.4	0.2 ± 0.1
A426G/N455I	1.0	ND	1.2 ± 0.3	0.5 ± 0.2	0.5 ± 0.1	ND	0.4 ± 0.2	10^−2^	0.7 ± 0.2	1.0 ± 0.1	0.2 ± 0.1
A426G/N508T	1.0	ND	1.2 ± 0.1	0.5 ± 0.3	0.5 ± 0.1	ND	0.6 ± 0.2	10^−2^	0.8 ± 0.1	1.1 ± 0.2	0.2 ± 0.2
A426G/T564R	1.0	ND	1.3 ± 0.3	0.4 ± 0.2	0.6 ± 0.1	ND	0.4 ± 0.1	10^−2^	0.7 ± 0.1	1.6 ± 0.3	0.2 ± 0.1
T443P	1.0	ND	1.1 ± 0.1	0.4 ± 0.1	0.7 ± 0.1	ND	0.5 ± 0.1	10^−2^	0.9 ± 0.1	1.3 ± 0.1	0.2 ± 0.1
N455I	1.0	ND	1.2 ± 0.2	0.4 ± 0.1	0.5 ± 0.1	ND	0.3 ± 0.0	10^−2^	0.6 ± 0.1	1.4 ± 0.3	0.2 ± 0.1
N455I/N556Y	1.0	ND	1.1 ± 0.5	0.3 ± 0.2	0.2 ± 0.1	ND	0.1 ± 0.0	10^−2^	0.7 ± 0.2	0.9 ± 0.3	0.2 ± 0.0
G585D	1.0	ND	0.9 ± 0.2	0.4 ± 0.2	10^−2^	ND	10^−2^	10^−3^	0.8 ± 0.2	1.1 ± 0.2	0.2 ± 0.1
S589A	1.0	ND	1.2 ± 0.3	0.1 ± 0.0	10^−2^	ND	10^−3^	10^−4^	0.8 ± 0.2	1.3 ± 0.2	0.2 ± 0.1

a Gray shading indicates low efficiency of plating values (≤10^−2^), with darker gray corresponding to lower efficiency. –, wild-type.

**FIG 3:**
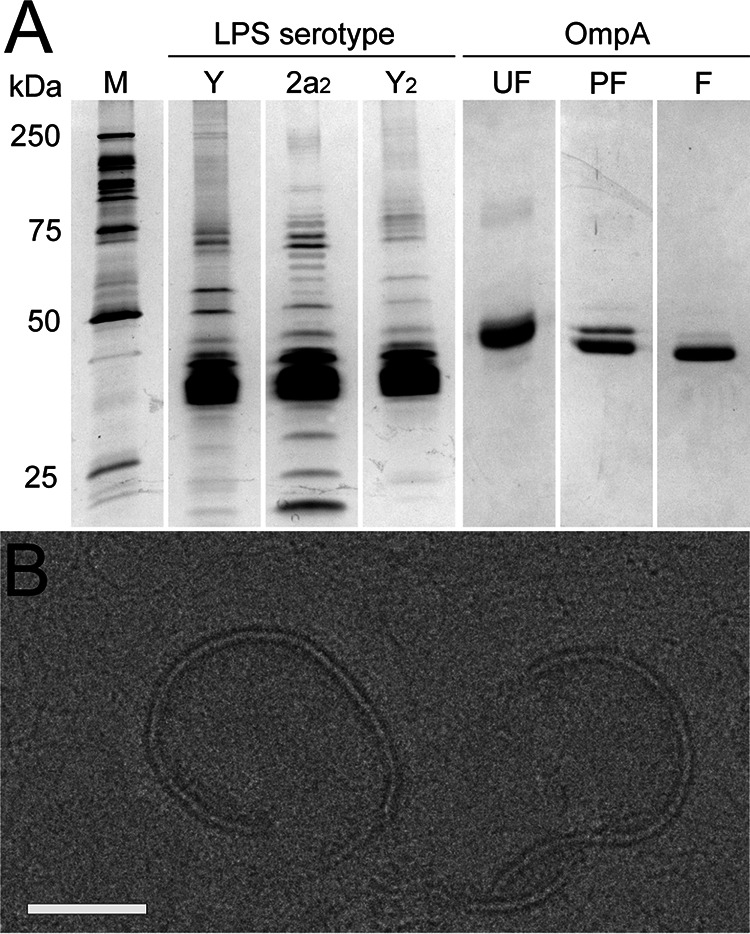
Components used for *in vitro* kinetics assays and visualization of interactions between Sf6 and LPS via cryoEM. Purity of samples was confirmed by acrylamide gel electrophoresis, followed by either silver (LPS) or Coomassie (OmpA) staining (A) and cryoEM (B). In A, the molecular weight marker (M) is shown on the left. For OmpA, three stages of folding are shown to demonstrate the difference between unfolded (UF), partially refolded (PF), and refolded (F) protein. In B, the scale bar corresponds to 50 nm.

**FIG 4:**
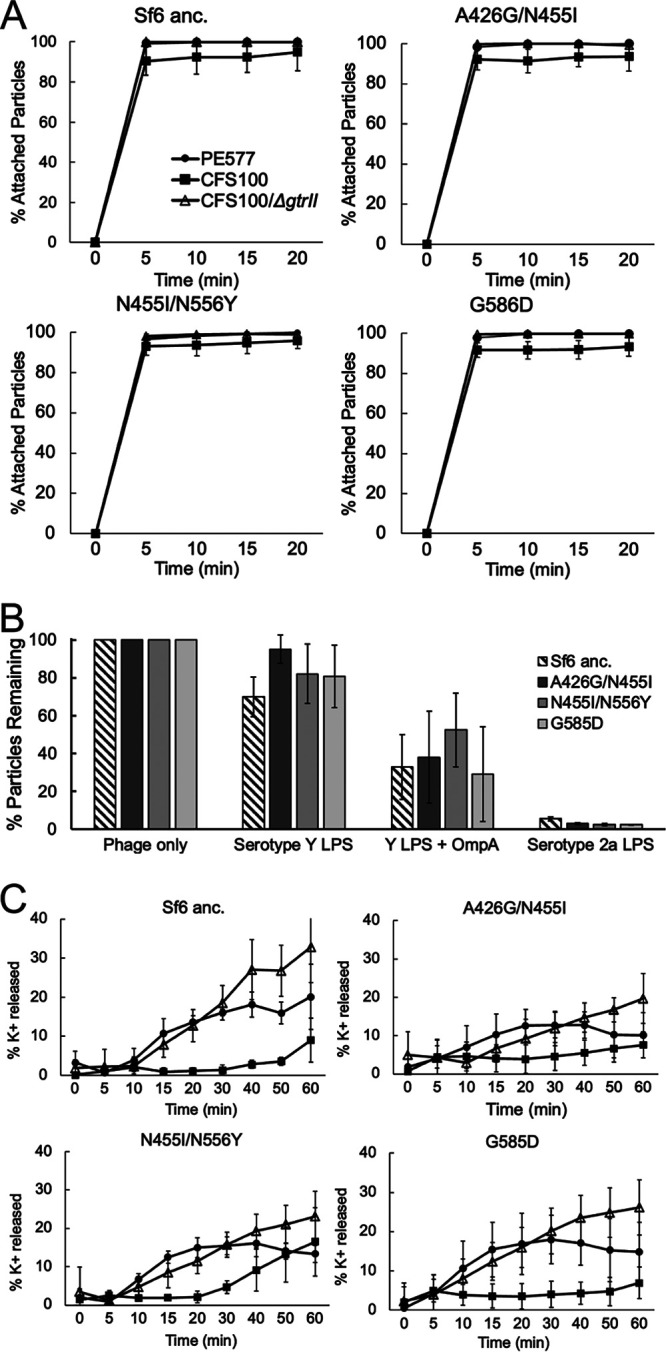
Kinetics of early infection stages of ancestral and evolved Sf6 strains. *In vivo* attachment kinetics (A), infectivity loss after incubation with purified LPS and/or OmpA (B), and potassium efflux kinetics (C) of ancestral Sf6 and three mutants.

Many phages, including Sf6, identify and infect their host using a two-step process: a reversible attachment phase, followed by an irreversible phage during which the genome is ejected. To determine which of these two stages might be hindered in the wild-type phage in the presence of CFS100 and how the altered tailspikes could overcome this block to access a new host, both attachment and ejection were investigated. The purity of cellular components was examined via SDS-PAGE (Fig. 3A) and cryoEM (Fig. 3B). LPS was silver stained and OmpA was stained via Coomassie at various stages of the refolding process prior to use in the in vitro assays. As shown in Fig. 4A, nearly all wild-type phage particles attach to the serotype Y host by 5 min. The same result was observed for the serotype 2a_2_ host. Attachment kinetics for three mutants, A426G/N455I, N455I/N556Y, and G585D, were measured to determine whether attachment was affected during experimental evolution. None of the three mutants exhibited altered kinetics versus the ancestral isolate. Thus, the rate of attachment does not explain why Sf6 is unable to infect CFS100, nor does it appear to contribute to the expanded host range phenotype.

### Incubation with serotype 2a_2_ LPS reduces Sf6 particle infectivity, but without genome ejection.

Next, genome ejection was initially examined *in vitro* using purified components. Phages were incubated at 37°C alone, with LPS, the secondary receptor OmpA, or with both LPS and OmpA. Plaque assays were then used to measure remaining PFU after the incubation period. Using this method, previous studies demonstrated that wild-type Sf6 requires both LPS and OmpA to ejects its genome *in vitro*, with LPS or OmpA alone being insufficient (22, 23). As expected, when incubated with serotype Y LPS alone, neither wild-type Sf6 nor the mutants showed significant loss of infectivity (Fig. 4B). Conversely, adding both serotype Y LPS and OmpA resulted in most particles losing infectivity, consistent with genome ejection observed in (23). Surprisingly, when particles were incubated with serotype 2a_2_ LPS alone, there was a complete loss of infectivity. As with our attachment assay, this result was observed for both the ancestral and experimentally evolved Sf6 isolates.

Infectivity loss could be explained by one of two phenomena: (i) *in vitro* genome ejection occurred, meaning the now empty phage particles are unable to infect subsequent host cells due to genome loss; or (ii) inhibition occurred, so the phage tail is blocked from binding to subsequent host cells, with no genome loss. The former case would suggest that irreversible binding between the tailspike and LPS is productive and could lead to a successful infection. The latter case would suggest that irreversible binding was nonproductive and did not lead to successful infection. To differentiate between these two possibilities, genome ejection was measured by potassium efflux. Upon penetration of the membrane and movement of the phage genome into the cell, potassium ions are released from the host and flow into the surrounding media: thus, measuring the change in potassium concentration serves as a more direct measure of genome ejection.

Ancestral Sf6 was incubated with serotype Y or serotype 2a_2_ host cells, and the change in potassium concentration was measured. As shown in Fig. 4C, incubation with serotype Y host cells induced greater potassium release than serotype 2a_2_ host cells. This suggests that even by 60 min, at which point most particles lose their infectivity when incubated with serotype 2a LPS, very few particles are ejecting their genomes in the presence of this host.

### Serotype 2a_2_ lipopolysaccharide appears to render Sf6 noninfectious by sticking to tailspikes.

**FIG 5:**
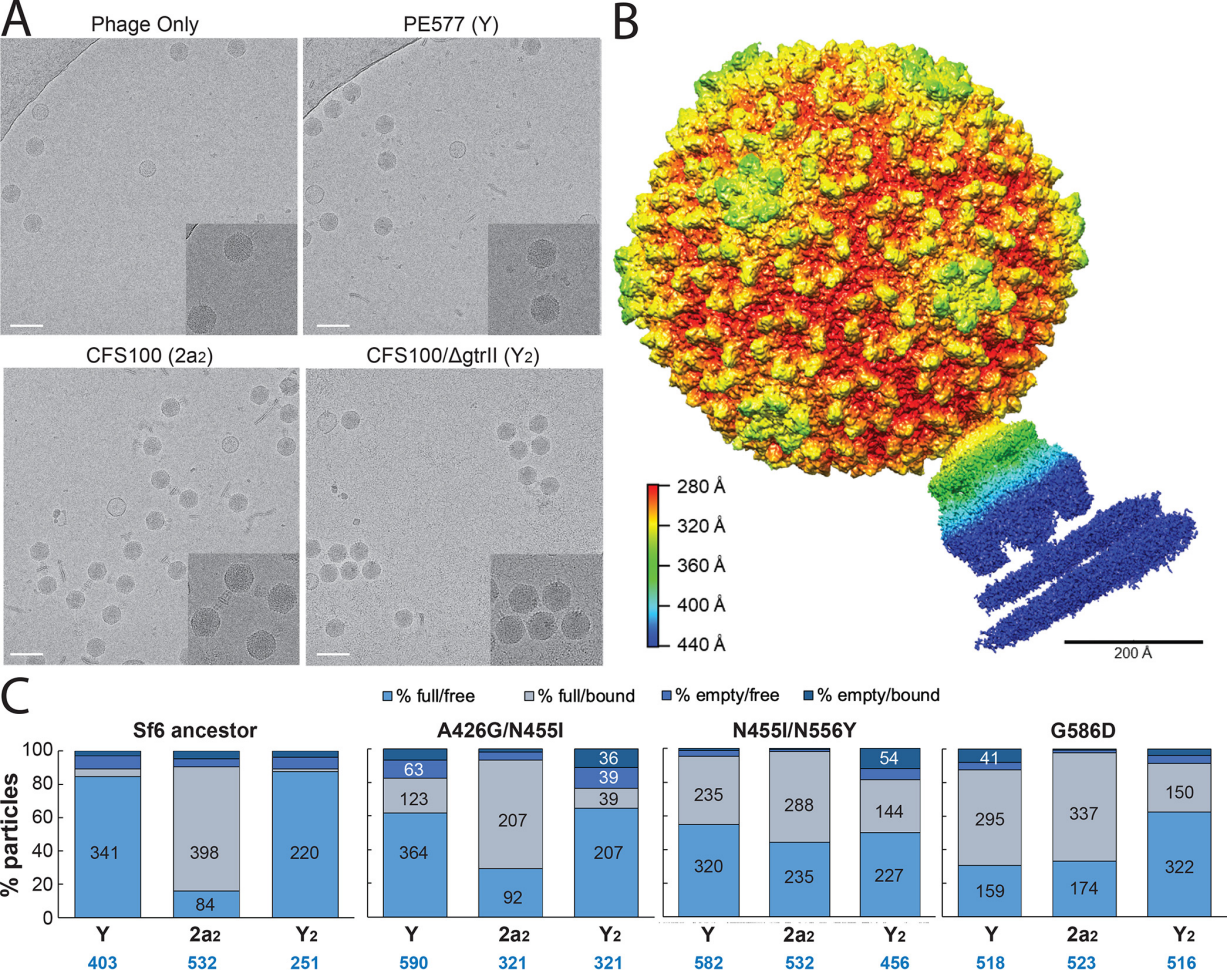
CryoEM analysis of Sf6 alone or in combination with purified LPS. (A) Representative images of the Sf6 ancestral strain: clockwise starting from top left without LPS, or with Y, 2a_2_, or Y_2_ types of LPS; insets show greater detail of particles; scale bar = 100 nm. (B) Asymmetric reconstruction of Sf6 particles bound to serotype 2a LPS at 5.1-Å resolution, colored by radial distance. (C) Manual assignment of particle status for Sf6 ancestral strain and three mutants based on their appearance: full/free, full/bound, empty/free, or empty/bound. Each category is represented as a percentage of all particles counted: the number of particles in each category is stated in the bar if possible; the total number of particles analyzed is indicated at the bottom in blue.

Since both ancestral and evolved Sf6 isolates showed reduced infectivity without genome ejection in the presence of LPS derived from the serotype 2a_2_ host, we hypothesized that the nonhost LPS was binding to phage particles more frequently or more strongly and resulted in inhibition of subsequent binding to susceptible host cells. To evaluate this possibility, the interactions between phage particles incubated with or without LPS were visualized directly. Aliquots from the *in vitro* infectivity loss assays were vitrified and examined by cryo-electron microscopy (cryoEM). Ancestral Sf6 and three mutants A426G/N455I, N455I/N556Y, and G585D were characterized under four conditions: phage alone, phage incubated at with serotype Y LPS derived from PE577, phage incubated with serotype 2a_2_ LPS derived from CFS100, or phage incubated with serotype Y_2_ LPS derived from CFS100/*ΔgtrII*. For each condition, approximately 500 particles were counted and scored as having: (i) an empty capsid, suggesting the genome had been ejected; (ii) a full capsid unbound to LPS; or (iii) a full capsid bound to LPS. Representative images of each condition are shown in Fig. 5A. For ancestral Sf6, particles incubated with serotype Y LPS were primarily in the “free and full” category regardless of whether the LPS was derived from PE577 or CFS100/*ΔgtrII*. Conversely, when phages were incubated with serotype 2a_2_ LPS, approximately 75% of the population were in the “bound and full” category. Combined with the potassium efflux data, this suggests the loss of infectivity is due to the phage tail proteins being stuck to LPS rather than the LPS inducing genome ejection.

Although our results indicated a block early in the infection process, the specific stage in the sequence of attachment and infection kinetics was unclear. Previous studies of the closely related phage P22 determined the series of events involved in phage attachment and genome ejection (14). Using cryo-electron tomography (cryoET), the authors showed that the phage particles initially bind the host at an angle, with two adjacent tailspikes bound to LPS and the tail needle in contact with the cell surface. This observed orientation of phage P22 matches our Sf6 mutant data, which show that the OmpC binding region is in the distal tail tip, where Sf6 is likely interacting with the cell surface in a similar fashion (see Fig. 1). The phage tailspikes then hydrolyze the O-antigen through a series of rapid release and rebinding events, bringing the rest of the tail closer to the cell surface. This is then followed by a reorientation of the particle, which positions the tail needle perpendicular to the membrane. The tail needle is then lost and the particle undergoes a dramatic rearrangement, which involves ejection proteins being released to form a tube. The genomic dsDNA is then translocated through this tube and into the host cell. The transition from oblique to perpendicular is inherently difficult to visualize but may involve additional tailspikes binding to and hydrolyzing nearby O-antigen molecules.

To determine how serotype 2a_2_ LPS may be inhibiting Sf6 particle infectivity, we performed an asymmetric reconstruction of the ancestral Sf6 strain bound to 2a_2_ LPS. If particles were stuck in the oblique orientation, we would expect to obtain a structure with the Sf6 tailspike bound at an angle, with contact between two tailspike trimers and the O-antigen of LPS. Conversely, if particles became stuck later, after reorientation, we would expect to see a perpendicular orientation, with the tail needle either present or absent (14). This orientation was observed in a study by McNulty et al. (37), using a mutant of phage P22 that lacked the tail needle protein. As shown in Fig. 5B, the particle appears to be oriented perpendicular to the LPS, with the tail needle intact and the particles retaining their genomes. Although this experiment was performed using purified components rather than whole cells, this may suggest LPS can trap the particle just before the tail needle is lost, possibly with all six tailspike trimers bound to the O-antigen of LPS.

For the mutants, although most particles were still in the “full and bound” category when incubated with the serotype 2a LPS, a significantly greater proportion were free from LPS (Fig. 5C). The three mutants analyzed also appeared to bind more frequently with serotype Y LPS when compared to ancestral Sf6, but this phenomenon did not transfer to the CFS100/*ΔgtrII* serotype Y_2_ LPS. Instead, in this last condition, the two double mutants A426G/N455I and N455I/N556Y were primarily empty particles, which may suggest altered genome ejection dynamics or stability in the presence of this type of Y_2_ LPS.

## DISCUSSION

**FIG 6:**
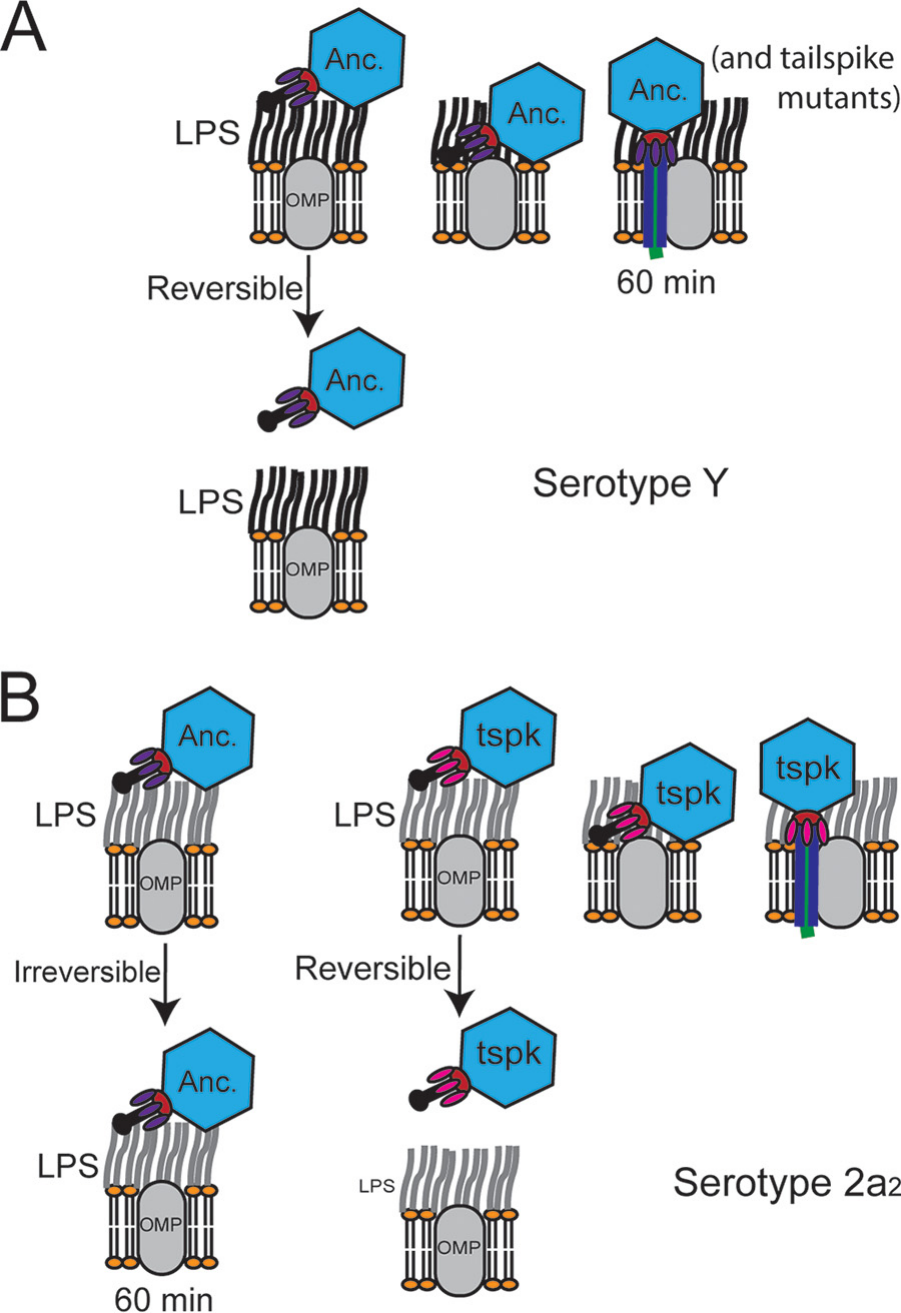
Proposed model of Sf6 interactions between S. flexneri serotypes Y and 2a_2_. (A) Both the ancestral (Anc.) and tailspike mutants (tspk) interact with serotype Y via reversible and productive interactions between the tailspike and LPS, followed by interactions with an outer membrane protein receptor (OMP). (B) The ancestral Sf6 interacts nonproductively with serotype 2a_2_ O-antigen via irreversible interactions, while the tailspike mutants are able to interact with serotype 2a_2_ similar to serotype Y.

Bacteriophage Sf6 undergoes a two-step attachment process to identify its host: an initial reversible attachment to its primary receptor LPS, and then a second, irreversible attachment to its secondary receptor OmpA or OmpC. Once irreversibly attached, the particle undergoes a rapid and drastic conformational change to translocate its genome across the membrane and into the host cell (Fig. 6A). The Sf6 tail complex is involved in each of these stages, and the tailspike is known to be critical for LPS binding and hydrolysis. The protein involved in irreversible attachment, however, remained unclear.

Here, we determined that the Sf6 gp14 tailspike can mediate interactions between both LPS and the secondary receptors OmpA and OmpC and that single point mutations in the *gp14* gene are sufficient to expand the Sf6 host range. Mutations near the LPS binding pocket may affect the strength of binding without affecting enzymatic activity of the protein. During preparation of this article, Teh et al. (38) also isolated an Sf6 mutant that could infect the serotype 2a_2_ host. This mutant had three mutations in the tailspike: Q325L, A426G, and N508T. Two of these mutations, A426G and N508T, were also isolated here. The authors had previously shown that even the ancestral Sf6 could hydrolyze a minimal amount of serotype 2a_2_ LPS (~20 to 30%) (38, 39), while the triple mutant could hydrolyze 68% of the O-antigen repeating units.

In a model where Sf6 does not infect serotype 2a_2_ hosts due to reduced interactions, or increased reversibility of reactions, between the tailspike and O-antigen, two observations may be expected: (i) incubation of phage and O-antigen would not affect particle infectivity; and (ii) phage particles and LPS would appear as separate and diffuse entities in cryoEM micrographs. In both cases, the opposite effects were observed here. This suggests an alternative model in which Sf6 cannot infect serotype 2a_2_ due to irreversible, nonproductive interactions rather than either reduced interactions or highly reversible ones (Fig. 6B). Glucosylated 2a and 5a O-antigens have previously been shown to assume a helical rather than a linear shape in solution or in a complex with antibodies (40, 41). Previous work using molecular dynamics demonstrated that mutations near the O-antigen binding site of the Sf6 tailspike can dramatically affect the flexibility of the site (42). As part of their study, Kunstmann et al. (42) modeled a T443C substitution. While we isolated T443P here, the effect of increasing flexibility may be similar. In this alternative model, we therefore hypothesize that mutations near the LPS binding site are decreasing the constraint of the O-antigen or altering electrostatic interactions between the tailspike and its receptors. These changes could allow for a better or a productive fit of the glucosylated 2a_2_ O-antigen structure to enable LPS hydrolysis (Fig. 6B).

While many mutants appeared to overcome the host barrier via altered LPS interactions, others showed a much greater reliance on the secondary receptors OmpA and/or OmpC. These mutations, which all mapped to the distal end of the tailspike, may increase the binding affinity for these secondary receptors as a way to compensate for slow or stalled LPS hydrolysis. Although these and several other isolates contained only single point mutations in their genomes, the frequency in which they arose was much lower than the point mutations in the A426G and N455I isolates. The variety of mutants recovered here may not have been identified in traditional single selection experiments. The G585D and S589A mutants had reduced plating efficiency on the CFS100 host compared to all other single or double mutants, suggesting this mechanism, while effective, may not be the most efficient.

Previous studies with closely related phage P22 demonstrate that the phage binds certain nonhost LPS weakly, allowing virions to detach from nonhost cells (30). By contrast, Sf6 appears to bind the serotype 2a_2_ nonhost LPS strongly, with virions less able to release from nonhost cells. It has also been shown that S. flexneri, like other enteric bacteria, alters LPS and/or Omp production depending on environmental conditions, such as temperature (35, 43). In freshwater reservoirs where S. flexneri persists, lower temperature would contribute to high levels of LPS production with low OmpC expression. In this case, phage may be more easily sequestered by LPS shed from the outer membrane of nonhost cells, with a lower chance of overcoming the LPS barrier through secondary receptor binding. One hypothesis is that this mechanism of phage sequestration may provide a community benefit for bacteria, where susceptible and resistant strains may c-exist, yet this idea remains to be experimentally confirmed. Conversely, in the warmer environment of the human intestine where S. flexneri causes disease, phage may be able to overcome these barriers due to lower LPS production and/or greater OmpC expression.

In the arms race between bacteria and phages, many well-studied mechanisms occur at the cell surface. These include modifications to the cell receptor or phage receptor-binding proteins (23, 44), reduced expression or shielding of receptors (45–47), and highly variable or horizontally transferred tail genes (48, 49). Outer membrane vesicles can also serve as decoys for bacteriophage (50, 51). Our results suggest that shedding LPS alone may also be sufficient to inactive bacteriophage in the environment, which would come at a greatly reduced resource cost. Using decoys rather than modifying receptor protein expression or structure is a method of herd immunity for bacteria. This mechanism can also reduce the size of the bacteriophage adaptive landscape by removing phage from the population. Since these decoys can sequester phage, it is important to determine how and when these decoys are most effective, and how phage can overcome this type of “passive” defense. In addition to understanding bacteria and bacteriophage ecology and evolution, it may be used to predict or inform the effectiveness of phage application for therapeutic use.

## MATERIALS AND METHODS

### Bacterial strains and plasmids.

The strains of bacteria used as hosts for Sf6, both PE577 and CFS100, are avirulent derivatives of S. flexneri and have been described previously (52–54). Mutant strains including *ompA::kan*, *ompC::kan*, *gtrII::kan*, or a combination thereof were generated using previously described methods (52) based on lambda red recombineering (55). For double mutants, the first kanamycin cassette was removed by transforming the strain with pCP20, which harbors Flp recombinase. After recovery, bacteria were incubated overnight with ampicillin at 30°C, and then single colonies were streaked onto a new plate. The next day, single colonies were chosen and incubated at 42°C to remove pCP20 and then screened for loss of the kanamycin cassette by PCR.

### Phage methods and experimental evolution scheme.

The ancestral isolate of Sf6 for this study is an obligately lytic mutant described in reference 55. This is a naturally occurring clear plaque variant of temperate Sf6 that was spontaneously induced from S. flexneri serotype 3a strain F3 and then recovered on serotype Y (32, 54, 56). It has been shown that ancestral Sf6 does not efficiently infect serotype 2a_2_ (32). Since the ability of Sf6 to infect CFS100 was very low (<10^−6^), we used a mixture of PE577 and CFS100 host strains during experimental evolution. Overnight cultures were therefore mixed in a ratio of 1:9 susceptible:nonsusceptible strains to maintain selective pressure while allowing the phage to replicate in a restricted number of host cells. To maximize our ability to screen replicate populations, a deep-well 96-well plate was used with a 0.5-mL total culture volume. At the beginning of each experiment, wells contained 450 μL Luria broth (LB), 30 μL of the 1:9 cell mixture, and 20 μL of a 10^6^ dilution of purified, isogenic Sf6, with a starting multiplicity of infection of ~0.1 phage per cell. This was then incubated at 37°C while shaking for 4 h for passage one. At the end of the incubation period, bacteria were lysed with chloroform and a replica pin tool was used to sequentially stamp 150 × 15-mm Petri plates seeded with nonsusceptible CFS100 and then the susceptible PE577 host cells. Debris were then allowed to settle in the 96-well plates and the lysates were stored at 4°C overnight. The following morning, this process was repeated using 20 μL of lysate from the previous day rather than the isogenic starting phage stock and fresh cultures of the host cells retaining the 1:9 ratio. If a clearing was observed around a pin stamp on the CFS100 screening plate, the experiment was stopped and the phage from the corresponding well was plaque purified.

Once a clearing was observed on CFS100, the phenotype was confirmed by plaque purifying on CFS100 at least three times. A single plaque was then picked and amplified in CFS100 to create an isogenic stock. Once cleared, the culture was centrifuged for 20 min at 6 000 × *g* to remove cellular debris, and then the supernatant was centrifuged for 90 min at 26 000 × *g* to pellet the phage. This pellet was then resuspended in phage dilution buffer (10 mM Tris, 10 mM MgCl_2_) by nutating at 4°C overnight. A final spin of 10 min at 6 000 × *g* removed any remaining cell debris, and the supernatant was retained as a high-titer phage stock.

To determine the genotype of mutant phages that could infect CFS100, genomic DNA was extracted from 1 × 10^10^ to 1 × 10^11^ phage particles using phenol-chloroform and sequenced at the Michigan State University (MSU) Research Technology Support Facility (RTSF). Sequences were aligned using Breseq as previously described (34).

### Attachment kinetics, *in vitro* infectivity loss, and potassium efflux assays.

Attachment assays were preformed as has been described previously (22). Briefly, phages were added to bacteria at a multiplicities of infection (MOI) of 10 and then incubated at 37°C. Aliquots of the mixture were taken at the time points indicated and centrifuged for 30 s to remove bacteria and any attached bacteriophages. The phages remaining in the supernatant were then quantified by plaque assay.

Infectivity loss assays were performed similar to *in vitro* genome ejection assays as previously described (23). Briefly, phages were incubated for 60 min in phage dilution buffer at 37°C either alone, with purified LPS at a final concentration of 0.5 mg/mL, or with purified LPS and OmpA refolded into Triton micelles at respective final concentrations of 0.5 and 0.05 mg/mL. At the end of the incubation time, the mixture was serially diluted in phage dilution buffer and infectious phage particles were quantified by plaque assay using the permissive host.

Potassium efflux assays were performed at 33°C. These were done by inoculating LB with a fresh overnight culture of bacteria and incubating with shaking to a concentration of 5 × 10^8^ cells/mL. The cells were then washed twice with fresh SM buffer (10 mM Tris, pH 7.5; 10 mM NaCl; 4 mM MgSO_4_) and resuspended in half the starting volume of SM buffer prewarmed to 33°C. After a 10- min equilibration stage, phages were added at an MOI of 5. The millivolt reading was measured using a Mettler-Toledo K+ selective ion electrode at time zero and then every 5 to 10 min afterwards. Standard curves for the electrode were conducted prior to addition of phage to determine accuracy of the electrode. The cells were lysed by boiling at the end of each experiment then allowed to cool. The values of potassium released based were calculated from the standard curve and normalized to the total amount of potassium released after lysis.

Results are presented with error bars representing the standard deviation for three separate experiments. Each experiment used a separate starting colony of the host strains. For the *in vitro* assays, LPS and OmpA were extracted from three biological replicates.

### Cryo-electron microscopy and data processing.

Small (3- to 5-μL) aliquots of virus/LPS mixtures were applied to R2/2 Quantifoil grids (Electron Microscopy Solutions) that had been glow discharged for 45 s in a Pelco Easiglow glow discharging unit. The samples were plunge frozen in liquid ethane using a Vitrobot Mark IV operated at 4°C and 100% humidity, with a blot force of 1 and 5 s of blotting time per grid.

For all virus/LPS mixtures, cryoEM data were collected at the RTSF Cryo-EM facility using a Talos Arctica equipped with a Falcon 3 direct electron detector, operating at 200 keV under low-dose conditions. Micrographs were collected at ×45,000 nominal magnification (2.24 Å/pixel) by recording 11 frames over 3 s for a total dose of 25 e-/Å^2^. For the Sf6-CFS100 LPS structure, cryoEM data Sf6-CFS100 LPS were collected at Purdue Cryo-EM facility using a Titan Krios equipped with a K3 direct electron detector and operating at 300 keV with a postcolumn GIF (20-eV slit width) under low dose conditions. Micrographs were collected at ×53,000 nominal magnification (0.816 Å/pixel) by recording 40 frames over 4.4 s for a total dose of 33 e-/Å^2^.

Dose-fractionated movies collected using the Falcon 3 direct electron detector were subjected to motion correction using the program MotionCor2. The resulting images were used for quantitative analysis of phage particles. Asymmetric reconstruction of Sf6 bound to CFS100 LPS was carried out using Relion 3.1.1. Briefly, the dose-fractionated movies were subjected to motion correction using Relion’s own implementation of MotionCor2. CTF estimation of the resulting images were estimated using CTFFIND-4.1 and particles were picked using the Autopick option. Then, 4× binned particles were extracted and subjected to 2D classification. A total of 18,964 particles were used for 3D refinement, with the Sf6 virion map (EMD-5730, scaled by a factor of 0.65 in EMAN2, Symmetrized using Relion 3.0.8) serving as an initial model. Refined particles were extracted again with 2× binning and subjected to 3D refinement. The overall resolution was estimated based on the gold-standard Fourier shell correlation = 0.143 criterion in the postprocess job.

### Data availability.

Final maps were deposited into the Electron Microscopy Data Bank (EMDB; accession no. EMD-26561).
